# Case-finding for COPD clinic acceptability to patients in GPs across Hampshire: a qualitative study

**DOI:** 10.1038/s41533-021-00216-0

**Published:** 2021-02-04

**Authors:** Danielle Rose, Emma Ray, Rachael H. Summers, Melinda Taylor, Helen Kruk, Mal North, Kate Gillett, Mike Thomas, Tom M. A. Wilkinson

**Affiliations:** 1 University Hospitals of Leicester, Leicester, UK; 2 NIHR ARC Wessex, Faculty of Health Sciences, University of Southampton, Southampton, UK; 3 University Hospital Southampton NHS Foundation Trust, Southampton, UK; 4 Faculty of Health Sciences, University of Southampton, Southampton, UK; 5 Department of Primary Care and Population Sciences, University of Southampton, Southampton, UK; 6 Faculty of Medicine, University of Southampton, Southampton, UK; 7 NIHR Biomedical Research Centre, University Hospitals Southampton NHS Foundation Trust, Southampton, UK; 8 Wessex Investigational Sciences Hub, University of Southampton Faculty of Medicine, Southampton General Hospital, Southampton, UK

**Keywords:** Chronic obstructive pulmonary disease, Health care, Medical research, Respiratory signs and symptoms

## Abstract

Despite high mortality and morbidity, COPD remains under-diagnosed. Case-finding strategies are possible, but patients’ perspectives are unexplored. Using qualitative methods, we explored the patient perspective of a case-finding intervention among at-risk patients in primary care. Semi-structured telephone interviews were transcribed and thematic analysis utilised. Seven patients without (mean age 64.5 years (58–74), *n* = 4) and 8 with obstructed spirometry (mean age 63.5 (53–75), *n* = 4) were interviewed. Themes identified were motives, challenges and concerns regarding attending the clinic. These included wanting to be well; to help with research; concern over negative impact to life from COPD diagnosis; perceived utility of the clinic; quality of information given; staff manner, approachability and knowledge; and perceived effects of the clinic on lifestyle, self-management and symptoms. The intervention was generally deemed useful and reassuring, although shared information was too detailed or irrelevant for some. Several reported positive lifestyle changes, improved symptoms and improved self-management.

## Introduction

Chronic obstructive pulmonary disease (COPD) is a leading cause of morbidity and mortality worldwide and its cost to the National Health Service, both direct and indirect, is substantial^1–3^. Within the past decade, hospital admissions for COPD exacerbations have increased by 50% and the annual cost of treating COPD has risen to £587 m^4–7^. In the UK, approximately 1.2 million people are currently diagnosed with COPD^5^. However, it is estimated that over half of individuals with COPD remain undiagnosed until significant disease has occurred^8,9^. A lack of public and clinical awareness of the indicators of COPD have been proposed as contributory factors to this delay in diagnosis, compounded by the slow, insidious onset of the disease. Consequently, patients often do not initially recognise their symptoms as abnormal and may wait until symptoms are more severe to seek medical advice^10–12^.

The early detection of COPD improves access to evidence-based treatments, such as smoking cessation, immunisation and pulmonary rehabilitation. These have the potential to improve quality of life and reduce morbidity and mortality rates, thus improving the financial burden to the health care system^13–15^. Actively case-finding patients with COPD above opportunistic and routine screening is endorsed by the Global Initiative for Obstructive Lung Disease, as well as many national health organisations^13^. A number of approaches have been trialled worldwide, including targeting ever-smokers (ex-smokers and current smokers) with symptom screening questionnaires and inviting those with positive respiratory symptoms to attend for diagnostic spirometry^16–23^. These methods are, in general, more effective at identifying new cases of clinically important COPD and cost less than routine care and screening.

To improve the process, risk-prediction models applied to routine collected data have been trialled^24,25^. A COPD risk score derived from a logistic regression model (the TargetCOPD trial) has been developed and validated in the UK from a retrospective cohort analysis, which compared case-finding methods to routine care in General Practice (GP)^25^. In this model, an algorithm comprising of age, smoking status, dyspnoea, prescriptions for salbutamol and prescriptions for antibiotics identified patients at high risk of undiagnosed symptomatic COPD and could be applied to routine clinical data in primary care.

While new methods to identify patients at risk of having COPD are proposed, encouraging these patients to attend for screening is key. Therefore, addressing potential barriers, challenges and concerns to patient uptake in GP may aid future case-finding implementation strategies. To date, only one research paper has explored the perceptions and experiences of patients attending a case-finding clinic while others have focussed on the views of health care professionals^26–28^.

Enocson et al. interviewed 40 participants who participated or declined to participate in the TargetCOPD study as well as those who DNA (did not attend)^26^. They found that barriers to attend case-finding clinics included the denial of symptoms, attributing breathlessness to age, stigma from the self-inflicted nature of the symptoms and respiratory symptoms not being their current health concern. In addition, physical barriers included lack of time to attend, inconvenience and the lack of feedback from the initial sreening^26^.

In the current qualitative study, we wanted to explore the perceived value and acceptability of the case-finding intervention among patients at risk of COPD who had been recruited into the ASSIST (A clinical interventional study into Airways diSease caSe-fInding and “At riSk” case management), which is currently in press and explained in the methodology section of the paper^29^. In the ASSIST study, the TargetCOPD algorithm was applied to primary care records and patients meeting an agreed threshold and deemed eligible were invited via the letter. Those wishing to attend booked an appointment at the case-finding clinic at their GP. At the clinic, trained respiratory nurse specialists, supported by respiratory physician colleagues from the University of Southampton Hospital Trust, obtained consent and a clinical history from the patient to evaluate whether lung disease was the primary cause of any presenting symptoms. This included an overview of respiratory symptoms, smoking history, family history of respiratory disease, exposures to fumes and chemicals and existing medical history, for example. In addition, patient-reported outcomes were obtained and quality-assured spirometry with reversibility was undertaken.

Bespoke health and symptom management advice was shared with patients attending the case-finding clinic where necessary, whether or not they were identified as having COPD. This might have included evidence-based information including smoking cessation or an inhaler technique check for those patients already using devices, in asthma for example. A summary report was provided for consideration by the GP, including treatment recommendations, further investigations and onward referral where there was likely to be a benefit. No further follow-up was provided by the study team as direct care needs remained with the patients’ designated GP.

In this qualitative study, we wanted to evaluate whether there were any concerns or challenges for patients attending these clinics. In addition, we wanted to explore how their needs were met in terms of delivery of health advice as part of the intervention, how that may have benefitted patients or not in terms of symptom reduction and to understand whether there were any further positive or negative consequences as a result of attending the clinic.

## Results

### Recruitment

**Fig. 1:**
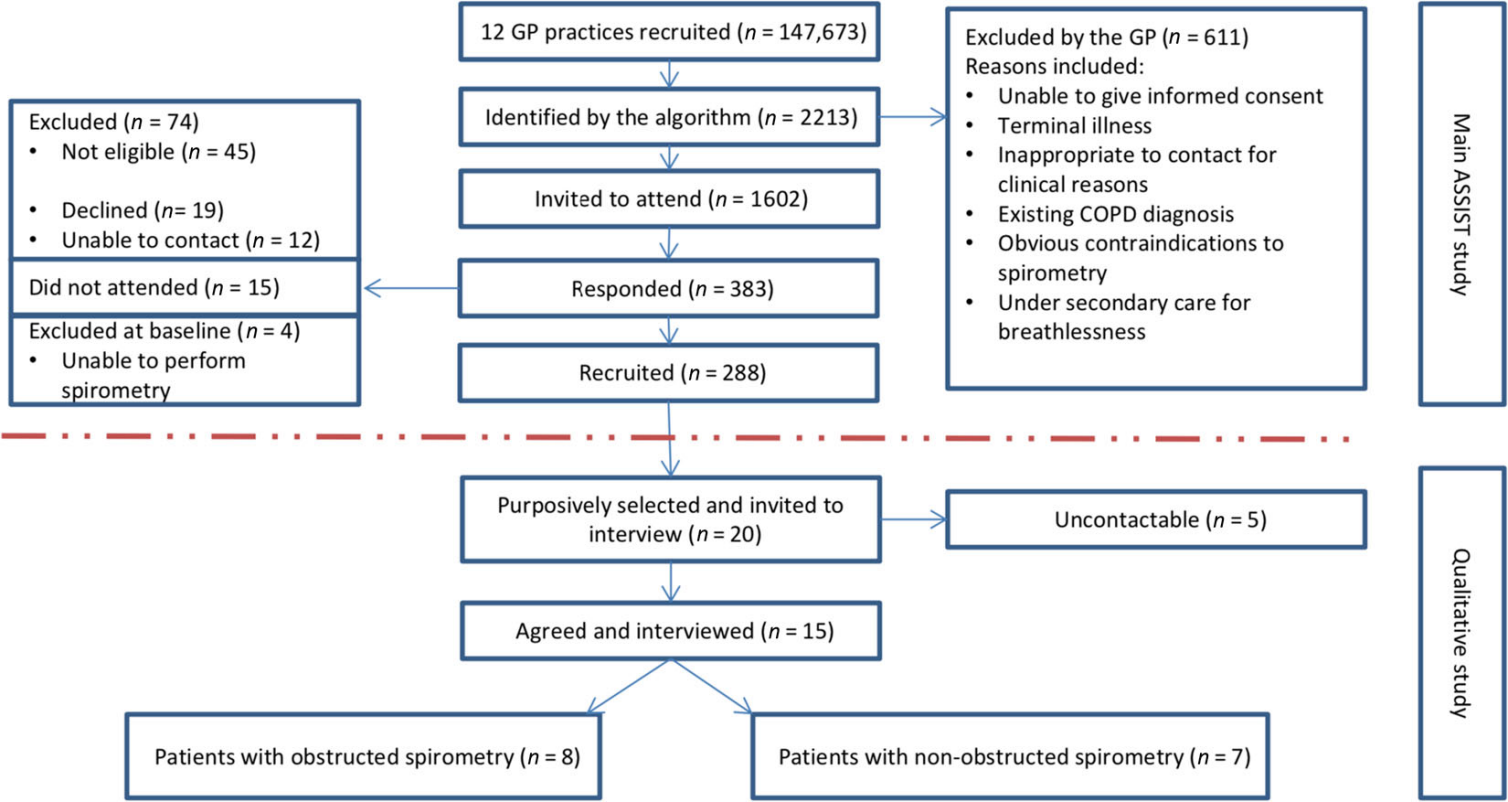
Recruitment flow diagram. Indicated is the recruitment of subjects in the ASSIST study. The diagram indicates the number of GPs patients were recruited from, number of patients identified by the algorithm, number invited to attend, number responded and number recruited. The purposeful selection of patients for the qualitative arm of the study is also indicated.

This study is nested in the ASSIST study, which was implemented in 12 GPs in Hampshire, UK, with a combined patient population of 147,673 (see Fig. 1)^29^. In total, 1602 patients were deemed eligible for the study and were sent a postal invitation from their GP to participate in the ASSIST study. Following further exclusions (e.g. not being able to complete spirometry) and taking into account patients who DNA, 288 (male 51%, mean age 63 years (SD = 6.71 years)) attended the case-finding clinic at their GP and consented to participate in the ASSIST study. In total, 76 (26.4%) patients who met the UK diagnostic criteria for COPD [post-bronchodilator airflow obstruction on spirometry (FEV_1_/FVC < 0.7), associated respiratory symptoms] were subsequently advised to see their GP in order to confirm any potential diagnosis and for symptom management.

Consent to participate in the qualitative study was taken at the same time as the main study, and ultimately, 15 patients were purposively selected and interviewed. Seven patients had no airflow obstruction and eight patients had obstructed spirometry and concurrent respiratory symptoms (suspected COPD). We included both categories of patients to gain a broad understanding of patient perspectives of the case-finding clinic whether or not there was any likelihood of respiratory disease. The second half of Fig. 1 shows more detail of the recruitment process into the qualitative sub-study, and Table 1 provides details of the patient characteristics.

**Table 1:** Patient characteristics.

Variable	Airflow obstruction, *n* = 8	Non-airflow obstruction, *n* = 7
Age (median, range)	63.5 (53–75)	64.5 (58–74)
Males	4	4
Caucasian	8	7
Asian	0	1
Current Smoker	0	1
MRC (median, range)	2 (1–4)	2 (1–2)
GOLD stage 1	3	0
GOLD stage 2	4	0
IMD decile (median, range)	7 (7–8)	8 (7–8)

*IMD* index of multiple deprivation (a weighted standardised measure of socioeconomic status ranging from 1 and area of high deprivation to 10 and area of low deprivations).

GOLD stage 1: Mild COPD FEV_1_ ≥80%; GOLD stage 2 FEV_1_ 50–79%.

**Fig. 2:**
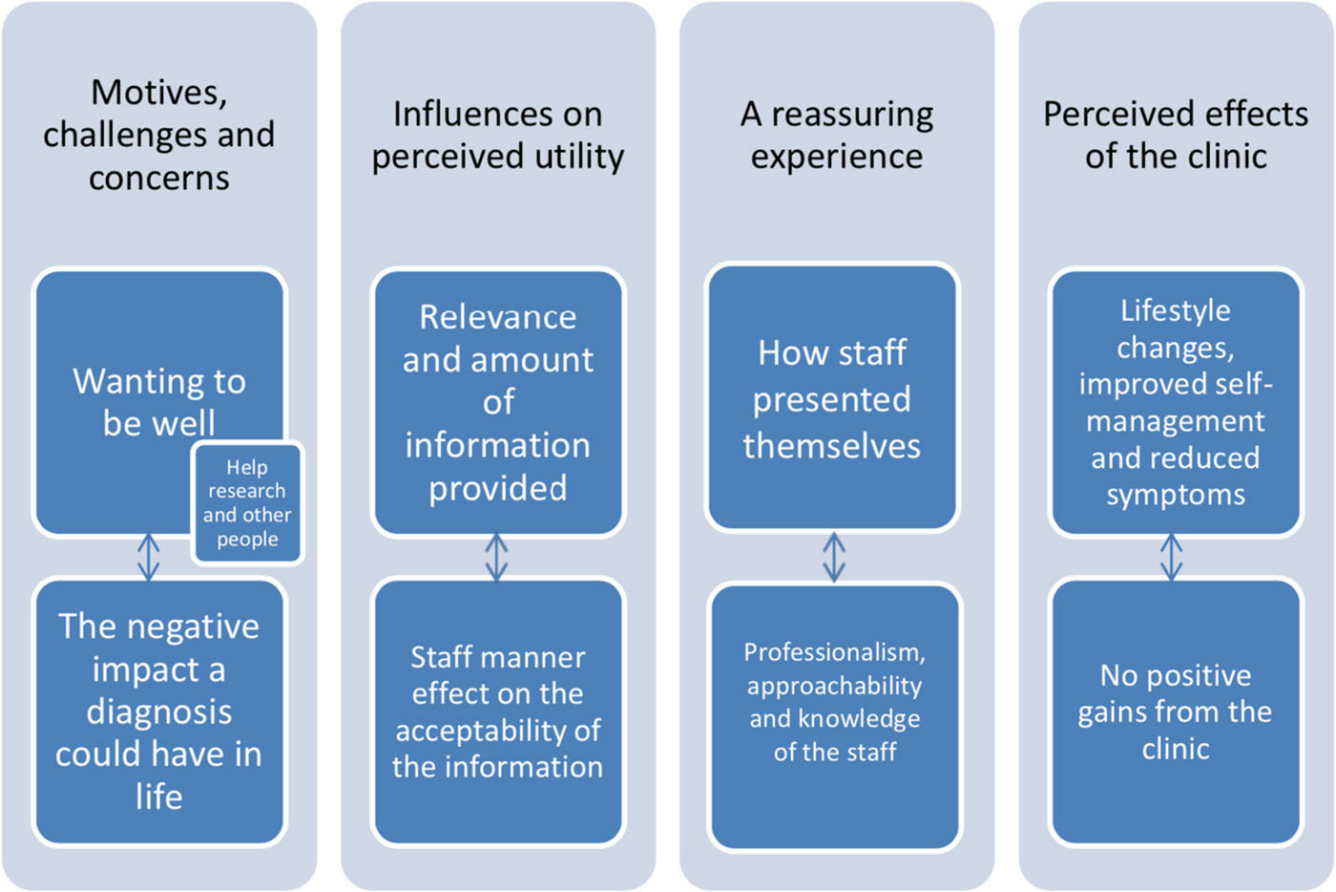
Overview of the themes and sub-themes presented in the data. Indicated are the themes and sub-themes of patients’ views on their experience of the clinic and its utility, which were discussed.

Regarding patient views on their experiences of the clinic and its utility, the following themes and sub-themes were discussed (Fig. 2).

### Motives, concerns and challenges to clinic attendance

This theme describes considerations, which influenced patients’ decisions to attend the clinic. Often this was a balancing act between weighing perceptions of the potential negatives associated with clinic attendance against the positives. A potential concern for patients was having a diagnosis that could reduce their quality of life. Specifically, (1) practical consequences, (2) the possibility of diagnosis reducing enjoyment of valued activities and (3) difficulty in denying a diagnosis if made.

“I feel I probably do have it, but I was quite happy for them to not put a name on it because it’s then in the background, it hasn’t gone away isn’t it? I know I am short of breath you don’t need to tell me that” [non-obstructed spirometry]

“This may seem obtuse, but I was looking for a diagnosis that gave me the most minimal risk in terms of travel insurance”. [obstructed spirometry]

Yet ultimately, patients with such concerns attended in the hope of being well, improving and/or reducing decline in their health. One account below provides an example of the considerations involved:

“I was really nervous and really anxious about it. Even when I rang up I was really anxious about it, thinking oh my gosh what if this goes against my job, what if this goes against going to [Country]. I did have a chat with different people on different occasions and they said ‘don’t be stupid the chance of you having to take oxygen with you away and to work is few and far between’. I thought about it really hard and thought it’s an opportunity to find out if there was anything seriously wrong with me before it’s too late. By coming in and doing these tests, has enabled me to find out well no actually you’re fine. But it’s only because you’re unfit.” [non-obstructive spirometry]

For some patients, altruistic reasons including the desire to help others and to aid research was described as being the main factor for clinic attendance. Some patients were also motivated to attend because someone was taking an interest in their health.

“I might be in poorer health in my older years. So, I thought if I can go through this and help someone else, that’s absolutely fine.” [obstructed spirometry].

*“*Oh fine it was a bit of a surprise, but I thought well it’s for research so I thought you know, I had done a few research things before because I have MS so, yeah. So, I thought you know, it’s good to help other people. So, I thought I would give it a go” [non-obstructive spirometry].

### Influences on perceived utility

This theme is about the acceptability of the clinics, the perceived usefulness of the information provided and what seemed to influence patient evaluations of clinic utility. Most patients reported that the information provided met their needs and was useful. These patients considered the information to be understandable and delivered at a suitable level.

“Looking back, I didn’t feel intimidated by the language that was used. If there was a word or phrases the nurse explained them all. Think there was one or two bits where I was like what does that mean? Ok great that’s fine. Or what are you going to do oh ok that’s fine. You know it was well explained and wasn’t too difficult to understand and the bit I had to read was explained to me. It was all explained very quickly and very easily.” [non-obstructive spirometry]

Patients also described attending the clinic with worrying symptoms that they were unsure how to manage. Verbal information given in the clinics was reported as lessening such concerns, improving patient understanding and giving them the tools to improve their symptoms and manage their existing lung disease.

“I feel better in myself knowing what’s wrong with me and what I can do to address it. When it flares up I know what to do. I know that I need to take my medication first thing in the morning and last thing at night. That’s what I wasn’t doing before. With a puff on my inhaler I know I will be fixed in five, so it’s definitely a positive experience for me.” [obstructed spirometry with a diagnosis of asthma]

“After the review I realised that things were not quite right with me. But now I realise it is something that can be relatively easily treated. Which is what I learnt from the program. The benefits of the drugs introduced to me, have had a positive impact on me. I have a better understanding of what I have got and why I have got it.” [obstructed spirometry]

However, not all patients found the clinic entirely useful. On these occasions, patients reported that the information provided was not relevant to them or that too much information was provided. It appeared for some patients that short factual information and reassurance would have been preferable over in-depth explanation.

“I was shown lots of charts and diagrams but, I am slightly colour blind and everything seems to be colour coded. So, I looked thinking ‘um yeah’. But you know it doesn’t bother me, I know there is this culture of let me tell you everything that could possibly go wrong. But all I want to know is am I going to be ok, I want reassurance.” [non-obstructive spirometry]

“I understood what they were talking about, but I couldn’t see how it referred to me half that time sort of thing.” [obstructive spirometry]

Despite this, only one participant did not feel she had received any benefit from attending the clinic. Other than the perception that the information given was too detailed, not reassuring enough and/or not relevant to the individual, there were no obvious differences demographically between the majority of participants who found the clinic useful and those who found it less so.

### A reassuring experience

This theme relates to how comfortable patients felt with staff and the level of reassurance they experienced. The majority of patients frequently reported that staff manner and how staff presented themselves was important. The key areas mentioned were (1) staff approachability, (2) staff professionalism and (3) staff knowledge. This was important to patients as it meant that their experience was one of reassurance and ease.

“The nurse had clearly done it before and clearly was not a novice in these sorts of things. Therefore, from my point of view, I was put at ease the whole time and was very comfortable about everything I was asked to do.” [obstructed spirometry]

It was also expressed that the holistic tailored approach taken by staff allowed them to express concerns outside of a respiratory scope. This appeared to make patients feel valued and increase the helpfulness of the clinic.

“I was having problem sleeping. So, I explained what happened and everything and the nurse said that it sounds like sleep apnoea. I was saying that my daughter has problems like that. Then I got talking about my daughter and things that happen with her and how I was dealing with it, because I nearly lost her and everything. The nurse was talking to me about things like that and it was really helpful and it didn’t seem all they were interested in was getting the test results” [obstructed spirometry]

### Perceived effect of the clinic

This theme is about how patients perceived the effect of clinic attendance. The majority of patients reported positive lifestyle changes following clinic attendance. Several patients described the changes that they had made, these ranged from weight loss, increased physical activity, quitting smoking and activities to improve their lung volume. However, for one patient attending the clinic had no impact on lifestyle and no changes were reported (Table 2).

**Table 2:** Patients’ perceived effect of clinic attendance.

Impact	Quote/evidence
Weight loss	“I mean I have lost some weight since I spoke to her. I listen to what she said and I have acted on it so.” [obstructed spirometry]
Stop smoking	“Have you made any changes to you smoking?” [Interviewer] “Well I have actually, that’s going to be it now I have made up my mind to call it a day. Which is good because it’s obviously going to benefit me so you have to take these roots don’t you? That is the right way to go.” [non-obstructive spirometry]
More health conscious	“I am more conscious, as I said earlier, I am a bit of a tinker. I like doing model making, which sometimes involves spray painting. You can understand that spray paints put a vapour in the air, so I am conscious now so I wear a mask. Also, we have done a lot of DIY around the house and I have never worn a mask, whereas now I do.” [obstructed spirometry]
Activities to increase lung volume	“Yeah it helped my lung capacity. I do it when no one is around not late at night because I would drive my husband mad. I am not singing professionally anymore, but I am singing at home which has helped.” [non-obstructed spirometry]
No impact	Following the clinic have you made any differences to your life? [Interviewer] “No nothing not really I will find out more when I see a doctor next week whether I do need any medication sort of thing and what the actual problem is.” [obstructed spirometry]

A positive staff manner through professionalism and approachability appeared to facilitate patients in making positive lifestyle changes. Patients appeared more amenable to listening and accepting of new information.

“It was more her attitude it was like two mates talking instead of a professional, a customer or whatever. When it’s like two mates talking, I think you listen more. I don’t like being told what to do it’s been a problem all my life. But when she spoke to me like that it’s not telling you what to do, she is just explaining why you should do it, if you see what I mean? I mean I have lost some weight since I spoke to her. I listened to what she said, and I have acted on it so.” [obstructed spirometry]

## Discussion

Patients with established COPD have previously been reported to have a poor understanding of disease trajectory and outcomes^1–3^. Enabling earlier access to smoking cessation support, initiation of therapy and timely referral to pulmonary rehabilitation may slow disease progression and decline of lung function plus reduce the need for hospitalisation^13–15^. Therefore, qualitative research that explores the motives, challenges and concerns to attend case-finding clinics as well as the acceptability of the intervention to patients may help improve patient uptake and assist with the early diagnosis of COPD.

Overall, patients deemed the case-finding intervention acceptable and beneficial, although one patient indicated that information provided was too detailed or perceived it as irrelevant to them. There were several underpinning themes that appeared to determine the success of case-finding clinics: (1) perceived utility, (2) staff manner, and (3) patients feeling reassured. Patients expressed that they felt respected, valued and listened to, which, for the most part, increased the acceptability of the information provided and increased the likelihood of them making positive lifestyle changes.

Our findings echo previous literature that found patients were largely supportive of case-finding clinics^26^. Patients presented with similar motives towards clinic attendance, including wanting to attend due to altruistic views, wanting to seek advice and wanting to feel well^26^. Key similarities in the concerns expressed were the fears of a potential diagnosis leading to financial ramifications and social stigmatisation^26^. Indeed, a patient in the current study indicated that they did not want a diagnostic label on their condition. The patient’s reasons were not explored and are therefore unclear why. However, when exploring the literature, it is plausible to consider several factors. A number of studies describe the social stigma associated with a diagnostic label of COPD leading to denial, low self-esteem and dis-engagement from social activities^30–32^. Patients may also have self-guilt and experience stigma from others as COPD is regularly perceived as a self-inflicted disease caused by smoking tobacco^30–32^. In addition, patients with COPD may face more stigma because their symptoms (dyspnoea, phlegm and cough) are more visible, in contrast to patients with well-controlled epilepsy or diabetes, for example^33,34^.

Although similar motives, challenges and concerns were reported for clinic attendance, contrasting views were expressed towards clinic implementation. In general, patients reported that the clinic met their needs in terms of the information provided being useful and understandable. It is possible that this was the case because patients who attended the case-finding clinic received a thorough health assessment with an experienced respiratory nurse specialist. The assessment included a broad range of strategies to improve respiratory symptoms and overall health whether or not related to COPD. The benefits to patients therefore may well extend further than respiratory outcomes, although, to date, other health outcomes have not been measured in the main ASSIST study^29^. In contrast, the TargetCOPD trial used a research assistant trained in spirometry to screen patients at the case-finding clinic and test results were fed-back to the patients’ GP to take action^24^. This screening method is faster and requires less training and therefore will allow for more patients to be seen. Furthermore, using respiratory nurses may ensure that patients gain timely health advice and feedback on their results.

While the majority of patients found the clinics valuable, leading to adaptations to lifestyle choices, some patients reported that the information provided was too detailed and was not perceived as being relevant to them. The specialist respiratory nurses may have felt the need to explain findings in detail particularly as the patients were research participants who were volunteering their time to take part and there were also less time constraints. Furthermore, the level of health literacy, both verbal and written, varies among individuals and is reported to be lower than average in patients with COPD^35^. Therefore, language should be adjusted and individualised so that patients can make informed decisions to be able to agree to treatment plans, which may improve their health outcomes in the longer term.

Having a good rapport with staff at the clinics appears important for initiating positive behaviour change and conveying important messages. Indeed, several patients reported increased confidence in self-managing their condition (e.g. asthma or COPD) following clinic attendance. This may be because the nurse-led clinic was structured to facilitate the promotion of self-care and self-management strategies, which is comparable to the findings of other studies that found positive practitioner–patient relationships improved patient outcomes, well-being, satisfaction levels and acceptability of the information provided^36–39^. One study explored the effects of nurse-led education cardiac clinics on patient survival and self-management^38^. It was shown that nurse-led clinics improved self-management at 3 and 12 months and reduced hospital admissions. Similar literature in patients with COPD supports these findings, showing increased patient knowledge about the disease, reduction in symptoms and increased physical activity^40–43^.

This study implemented a careful and robust design. Multiple peer reviews, multiple coding and patient and public involvement (PPI) was used to critically appraise the methodological design and data analysis, improving credibility and dependability.

During patient interviews, similar views and experiences were articulated indicating data saturation, suggesting that the sample size was appropriate for the study. Although purposeful sampling was used to achieve heterogeneity, this was difficult to achieve. Indeed, most patients were Caucasian with English as their first language. An individual’s ethnic and cultural background may influence how an intervention is perceived. Therefore, a more multicultural sample may show differences in the views and experiences obtained.

The reasons behind non-clinic attendance were not investigated. These were patients who received an invitation to attend the case-finding clinic but decided not to participate for unknown reasons. The study team did not have permission to contact these patients. This may mean that the information presented only applies to those who responded to invitation and cannot be extrapolated to the large number of patients who did not respond.

It is possible that patients who agreed to participate in the study may have been persuaded by receiving a formal direct invitation from their GP and influenced by the state of their relationship with their doctor and GP leading to possible unintentional bias. Patients who had a positive experience of the case-finding clinic may also be more willing to participate in this study and therefore be biased in favour of the intervention. Indeed, this study presents an overview of the experience of patients attending a specific case-finding clinic and the findings may not be generalisable to other case-finding clinics in different geographical areas and populations.

Further research investigating the views and experiences of patients attending case-finding clinics is needed to determine the best approach, whether this be specialist team or GP led for example. This should include an analysis of short- and long-term follow-up to determine the impact of the intervention. This study highlighted that some patients found the information provided difficult to understand or irrelevant to them. More research would be beneficial on health professional adaptations to different levels of health literacy in patients with COPD in case-finding clinics. Future research should also aim to gather the views of patients with different ethnic backgrounds and those who did not attend the clinic. This may reveal other existing motives, barriers, concerns and challenges to attending case-finding clinics that were not captured.

In conclusion, patients who responded to the invitation found the case-finding intervention to be acceptable and useful when it made a positive impact on their lives in some way. Percieved benefits included receiving reassurance that their lungs were healthy, being given information on how to maintain their health or their condition and having a discussion on how to better manage any symptoms. Although some patients felt that the information was difficult to understand or not relevant to them, it appears that the way that case-finding clinics were delivered, specifically the ability of the clinician to tailor information and the relationship formed between patient and practitioner, may influence a patient’s willingness to make important lifestyle changes, potentially impacting on the long-term trajectory of their health.

## Methods

### Ethical approval

Ethical approval and protocol approval for the ASSIST study and the qualitative sub-study was provided by Southampton B Ethics Committee (16/SC/0629), all relevant ethical regulations were complied with and the trial is registered on ClinicalTrials.gov (ID: NCT03355677). During the planning phase, PPI was provided by two members of the public, both of whom were living with chronic lung conditions, and advised on the research design, interview questions and interview technique. Informed consent was obtained from all human participants who took part in this study.

### Recruitment for interviews

At the case-finding clinics, patients willing to participate in the qualitative study provided written informed consent to be contacted by the qualitative researcher at a later date. A sample of patients were chosen to achieve heterogeneity using a specific sampling strategy (described below). They were then contacted via telephone by the main author who was conducting the interviews to establish their interest and have any questions addressed. Once the information was fully understood and the study process was agreed, interview dates were mutually arranged. Audio-recorded informed consent was obtained on the day of the interview, prior to the interview commencing.

### Eligibility criteria

All participants (*n* = 288) recruited to the ASSIST study were eligible to participate in this research^26^. The eligibility criteria for the ASSIST study is presented in Table 3. For recall purposes, participants needed to have attended the case-finding clinic no longer than 3 months prior to the interview date^44^.

**Table 3:** Patient eligibility criteria for the ASSIST study.

Inclusion criteria	Exclusion criteria
• Registered with one of the participating GPs as of 1 January 2015	• Unable to give informed consent
• Age 40–79 years	• Existing or previous COPD diagnosis
• Ex-smoker or current smoker	• Under secondary care for investigation of breathlessness algorithm
• No COPD diagnosis	• Patients whom the GP or clinical investigator deem inappropriate to participate
• Willing and able to give written informed consent	• Suffering from a terminal illness
• Identified by a Read-code-based computer algorithm using the following factors: smoking history, evidence of respiratory symptoms, salbutamol usage, and antibiotic usage for respiratory issues^15^. Patients who met an agreed threshold with a greater chance of having COPD and were invited to participate in the ASSIST study	• Obvious contraindications to spirometry (e.g. unstable abdominal aortic aneurysm)

### Sampling strategy and sample size

The sample was chosen to maximise heterogeneity. Sequential sampling based on age, gender and deprivation scores (index of multiple deprivation) was used to identify a broad range of potential participants. In addition, equal numbers of patients with and without airflow obstruction were (FEV_1_/FVC ratio <0.70) invited to participate to allow for more diversity. Fifteen patients participated in this study, which enabled thorough probing of additional themes so that data saturation was achieved into the phenomenon of interest^45,46^.

### Study design

A qualitative semi-structured interview design was utilised, as this allowed patient perceptions and experiences to be explored in depth^47^. The researcher undertook the study from a subtle realist, pragmatic standpoint. Subtle realism is an accepted perspective in health research and, in this case, was appropriate to the study design^48^. It also reflects the researcher’s own position that an external reality exists independent of observers but can only be accessed via the perceptions of individuals and the ways in which they are interpreted. The researcher aimed to explore the views of participants as fully as possible, while recognising that the resultant understanding could approximate but never *exactly* reflect that of the participants^48^. Pragmatism, where the research method is chosen based on the ability to address a research question, was adopted as the impetus for the study and was driven by a real-world clinical problem, rather than a theoretical concept^48,49^.

### Data collection

Data was collected via semi-structured telephone interviews, which the researcher conducted in a private room to protect confidentiality. Telephone interviewing was selected versus face-to-face interviewing, as it removed any travel-related barriers to participation and had the potential to promote participant candour^50^. Interviews followed an interview guide (Table 4) and lasted approximately 25 min (median = 24.5 min). The interview guide was devised with support from PPI and the supervisors to allow for the research aims to be fulfilled^51^. Patients were asked if they had any concerns after receiving a letter from the GP asking them to participate in a study because they had been identified as being at risk of having COPD and what motivated them to attend the clinic. Patients were also questioned on their experience of attending the case-finding clinic, what they liked and did not like and what they thought of the information provided. In addition, patients were asked whether there had made any lifestyle changes after attending the case-finding clinic. Both open questioning and flexible sequencing were used to facilitate the building of rapport between the interviewer and interviewee, allowing for greater depth of information about the utility of the clinic through free-flowing speech^52^.

**Table 4:** Interview guide.

(1) When you were first invited to the clinic, can you tell me how you felt?
(2) Can you tell me what made you decide to attend the clinic?
(3) Can you tell me about your experience at the clinic?
(4) Was there anything you liked or disliked about the clinic?
(5) As part of the clinic, there is often quite a lot of information provided.
Can you tell me about the information you received and how understandable it was for you?
(6) Following the clinic can you tell me any ways that it made a difference to your life?
• Is there anything that we have not spoken about that you would like to talk about?

The main author, who is female, conducted the interviews as part of a thesis for a MSc in Physiotherapy at the University of Southampton where she received in-depth research training to undertake the project. Supervision was provided by experienced researchers at the University (E. Ray, R. Summers, and M. Taylor) who conducted peer reviews at multiple points to enhance confirmability and rigour. The interviewer was not known to the patients as she did not work in the case-finding clinics and the study nurses would have explained the project during the consent process. All interviews were recorded using a digital recorder and transcribed verbatim. Key points and reflections were recorded after each interview. All transcripts were anonymised using pseudonyms and identifiable information was removed to maintain participant confidentiality.

### Data analysis

Thematic analysis was used to analyse the data. From initial reading, the semantic themes, those views and experiences explicitly identifiable in the transcripts, were noted. The transcripts were then read repeatedly and reflectively to enable deeper familiarisation, from which patterns and relationships emerged; the latent underlying sub-themes from which a more meaningful understanding could be achieved^53,54^. Recurrent concepts were manually grouped into themes and sub-themes. This was continued until no further new information was developed, and the analysis was considered to be a full description of the similarities and differences in patients’ experiences across the data^55^. During analysis, the data were peer review coded to (1) help ensure connections in the data were not missed and (2) support the first author’s reflexivity, by challenging any assumptions and, in so doing, reducing the impact of any one individual’s personal biases^55^.

### Reporting summary

Further information on research design is available in the Nature Research Reporting Summary linked to this article.

## Supplementary information

Reporting Summary

## Data Availability

All data generated or analysed during this study are included in this published article.
