# Supplementary material for: Case-finding for COPD clinic acceptability to patients in GPs across Hampshire: a qualitative study
Source: NPJ Prim Care Respir Med. 2021 Feb 4;31:4. doi: 10.1038/s41533-021-00216-0 (PMC7862661; doi:10.1038/s41533-021-00216-0)
Supplement: Supplementary file 1 — Reporting Summary [file 41533_2021_216_MOESM1_ESM.pdf]

nature research

Corresponding author(s): Tom Wilkinson

Last updated by author(s): Sep 15, 2020

Reporting Summary

Nature Research wishes to improve the reproducibility of the work that we publish. This form provides structure for consistency and transparency in reporting. For further information on Nature Research policies, see our Editorial Policies and the Editorial Policy Checklist.

Statistics

For all statistical analyses, confirm that the following items are present in the figure legend, table legend, main text, or Methods section.

n/a	Confirmed	
☐	☒	The exact sample size (*n*) for each experimental group/condition, given as a discrete number and unit of measurement
☒	☐	A statement on whether measurements were taken from distinct samples or whether the same sample was measured repeatedly
☒	☐	The statistical test(s) used AND whether they are one- or two-sided<br>*Only common tests should be described solely by name; describe more complex techniques in the Methods section.*
☒	☐	A description of all covariates tested
☒	☐	A description of any assumptions or corrections, such as tests of normality and adjustment for multiple comparisons
☒	☐	A full description of the statistical parameters including central tendency (e.g. means) or other basic estimates (e.g. regression coefficient) AND variation (e.g. standard deviation) or associated estimates of uncertainty (e.g. confidence intervals)
☒	☐	For null hypothesis testing, the test statistic (e.g. *F*, *t*, *r*) with confidence intervals, effect sizes, degrees of freedom and *P* value noted<br>*Give P values as exact values whenever suitable.*
☒	☐	For Bayesian analysis, information on the choice of priors and Markov chain Monte Carlo settings
☒	☐	For hierarchical and complex designs, identification of the appropriate level for tests and full reporting of outcomes
☒	☐	Estimates of effect sizes (e.g. Cohen's *d*, Pearson's *r*), indicating how they were calculated

Our web collection on statistics for biologists contains articles on many of the points above.

Software and code

Policy information about availability of computer code

Data collection	N/A a previously published algorithm was used:<br>Haroon S, Adab P, Riley RD, Fitzmaurice D, Jordan RE. Predicting risk of undiagnosed COPD: development and validation of the TargetCOPD score. The European respiratory journal. 2017;49(6).
Data analysis	N/A a previously published algorithm was used:<br>Haroon S, Adab P, Riley RD, Fitzmaurice D, Jordan RE. Predicting risk of undiagnosed COPD: development and validation of the TargetCOPD score. The European respiratory journal. 2017;49(6).

For manuscripts utilizing custom algorithms or software that are central to the research but not yet described in published literature, software must be made available to editors and reviewers. We strongly encourage code deposition in a community repository (e.g. GitHub). See the Nature Research guidelines for submitting code & software for further information.

Data

Policy information about availability of data

All manuscripts must include a data availability statement. This statement should provide the following information, where applicable:

- Accession codes, unique identifiers, or web links for publicly available datasets
- A list of figures that have associated raw data
- A description of any restrictions on data availability

This has been include within the manuscript which reads as "All data generated or analysed during this study are included in this published article [and its supplementary information files]."

Field-specific reporting

Please select the one below that is the best fit for your research. If you are not sure, read the appropriate sections before making your selection.

☒ Life sciences ☐ Behavioural & social sciences ☐ Ecological, evolutionary & environmental sciences

For a reference copy of the document with all sections, see nature.com/documents/nr-reporting-summary-flat.pdf

Life sciences study design

All studies must disclose on these points even when the disclosure is negative.

Sample size	Sample Size is clearly outlined in Figure 1
Data exclusions	We refer the editor to Figure 1 for comprehensive details on data exclusion
Replication	This was a case finding study and at risk participants were identified using an algorithm.
Randomization	This is not applicable to this study as it was a case finding study and not a randomized trial.
Blinding	This is not applicable to this study as it was a case finding study and not a randomized trial.

Reporting for specific materials, systems and methods

We require information from authors about some types of materials, experimental systems and methods used in many studies. Here, indicate whether each material, system or method listed is relevant to your study. If you are not sure if a list item applies to your research, read the appropriate section before selecting a response.

Materials & experimental systems

n/a	Involved in the study	
☒	☐	Antibodies
☒	☐	Eukaryotic cell lines
☒	☐	Palaeontology and archaeology
☒	☐	Animals and other organisms
☐	☒	Human research participants
☐	☒	Clinical data
☒	☐	Dual use research of concern

Methods

n/a	Involved in the study	
☒	☐	ChIP-seq
☒	☐	Flow cytometry
☒	☐	MRI-based neuroimaging

Human research participants

Policy information about studies involving human research participants

Population characteristics	Study eligibility criteria included being registered with the participating practices from 1st January 2015 or before, age ≥40 and ≤79 years, no previous diagnosis of COPD, smoker or ex-smoker and able to complete spirometry testing.
Recruitment	. In total, 12 local General Practice surgeries agreed to participate in the programme from rural and urban settings and a broad range of deprivation profiles. The practices agreed for the team to apply the algorithm to the electronic patient medical records, to send mailed invitations for screening at the surgery for those identified as being at risk, to provide use of their premises for the diagnostic sessions and to receive information and recommendations from the outreach team.
Ethics oversight	Ethics approval was provided by Southampton B Ethics Committee (16/SC/0629), and the trial is registered on ClinicalTrials.gov (ID: NCT03355677).

Note that full information on the approval of the study protocol must also be provided in the manuscript.

Clinical data

Policy information about clinical studies

All manuscripts should comply with the ICMJE guidelines for publication of clinical research and a completed CONSORT checklist must be included with all submissions.

Clinical trial registration	N/A
Study protocol	This was a case finding study and a study protocol was not published

Data collection	Recruitment occurred between January 2017 and January 2018. Those attending were followed up with postal questionnaires at 3 and 12 months.
Outcomes	In this qualitative study we wanted to evaluate whether there were any concerns or challenges for patients attending these clinics. In addition, we wanted to explore how their needs were met in terms of delivery of health advice as part of the intervention, how that may have benefited patients or not in terms of symptom reduction and to understand whether there were any further positive or negative consequences as a result of attending the clinic. Patients were asked if they had any concerns after receiving a letter from the GP practice asking them to participate in a study because they had been identified as at risk of having COPD and what motivated them to attend the clinic. Patients were also questioned on their experience of attending the case-finding clinic, what they liked and didn't like and what they thought of the information provided. In addition, patients were asked whether there had made any lifestyle changes after attending the case-finding clinic. Both open questioning and flexible sequencing were used to facilitate the building of rapport between the interviewer and interviewee, allowing for greater depth of information about the utility of the clinic through free-flowing speech.
